# Comparisons of pulsed ultrasound‐assisted and hot‐acid extraction methods for pectin extraction under dual acid mixtures from onion (*Allium cepa* L.) waste

**DOI:** 10.1002/fsn3.3657

**Published:** 2023-09-07

**Authors:** Emine Şen, Ersen Göktürk, Vugar Hajiyev, Erdal Uğuzdoğan

**Affiliations:** ^1^ Pamukkale University Department of Chemical Engineering Denizli Turkey; ^2^ Hatay Mustafa Kemal University Department of Chemistry Hatay Turkey

**Keywords:** acid mixtures, high methoxy, hot‐acid extraction, onion waste, pectin, pulsed ultrasound‐assisted extraction

## Abstract

The aim of this study is to compare the physicochemical properties and yields of pectins extracted from onion waste under hot acid (HAE) and pulsed ultrasound‐assisted extraction (PUAE) methods using different organic–inorganic acids, their mixtures, and pure water. The extraction temperature for experiments carried out under HAE was kept at 90°C for 90 min, whereas PUAE experiments were accomplished at RT in 15 min. In general, HAE gave better pectin yields compared with PUAE due to the significance of the increasing extraction temperature for the release of pectin from the plant matrix. While the maximum pectin yield from onion waste was 16.22% for HAE, the highest yield for PUAE was 9.83%. PUAE provides less time‐ and energy‐consuming extraction of pectin within 15 min and thus seems to be more economic compared with the HAE. According to the physicochemical properties (equivalent weight (EW), degree of esterification (DE), methoxyl (MeO), and galacturonic acid (Gal‐A) contents) of obtained pectins, extracted pectins were mostly high methoxy pectin. While the DE and MeO values of pectins extracted in organic acid conditions under HAE were higher, these values were found to be higher for pectins extracted in inorganic acids under PUAE. For acid mixtures, the DE and MeO values of pectins under HAE were mostly found to be lower than those under PUAE. Sequential PUAE and HAE methods for the extraction of pectin from onion waste were also found to be useful in terms of obtaining higher yields and better physicochemical properties. The highest pectin yield was 20.32% for the sequential PUAE and HAE methods. FT‐IR analyses of the extracted pectins by both HAE and PUAE methods showed similar vibration bands compared with those of commercial citrus pectin.

## Graphical abstract

Pectin extraction under pulsed ultrasound‐assisted and hot‐acid extraction methods in dual acid mixtures from onion waste.

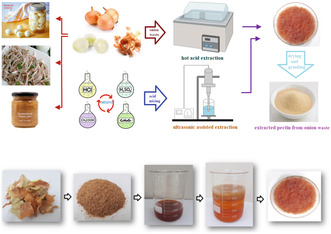

## 1 INTRODUCTION

The growing world population and changes in consumer behavior result in an increase in industrial food production (Chandrasekaran, 2013). Food waste is one of the most serious concerns for the food processing industry due to the loss of valuable nutrient sources. The food industry produces a variety of waste with different compositions and structures depending on the raw material and the process technology. Agro‐industrial residues usually consist of peels, shells, skins, stems, straws, seeds, pomace, and other non‐edible plant parts (Chandrasekaran, 2013; Galanakis, 2012). Nowadays, agricultural and industrial by‐products have gained more interest due to their potential values (Galanakis, 2012; Matharu et al., 2016). Low cost, abundance, availability, and renewability are the main advantages of agro‐industrial waste as secondary sources for valuable constituents (Cebin et al., 2020).

World vegetable production increased from 682 million tons to 1.128 billion tons between 2019 and 2020 due to the growing population and increasing demand. With a 9% market share in total vegetable production, onion was the second most harvested vegetable after tomato in 2020 (“World Food and Agriculture Statistical Yearbook 2021,” 2021). China, India, Pakistan, the United States, and Turkey are the main countries for onion production. Onion (dry) production in Turkey was around 2.28 million tons in 2020 (“Food and Agriculture Organization of the United Nations,” 2023). Approximately 10% of onion waste, consisting of skin and inedible parts, is generated during the processing of dried onions or its use in restaurants. Approximately 113 million tons of onion waste are produced around the world every year, considering that 10% of the total harvested onion is thrown away as waste.

Onion waste is produced in large quantities, consisting of onion skins, roots, and other waste parts (El Mashad et al., 2019; Sharma et al., 2016). Onion waste cannot be used as fodder or organic fertilizer due to its specific flavor or possible toxicity. The composting of onion waste is limited due to its high contents of sulfur‐containing compounds. It is also expensive to destroy onion waste by combustion due to its high moisture content (Benitez et al., 2011, 2012; Schieber et al., 2001). Industrial production of onion waste has increased due to the enhanced demand for onions in the global market in recent years. The increasing amount of onion waste and the difficulties in the conventional disposal of this waste result in environmental concerns (Cebin et al., 2020). Onion waste can be used as a source of food components due to their rich components such as dietary fiber, fructooligosaccharides, flavonoids, and alk(en)yl cysteine sulfoxides (Griffiths et al., 2002). Due to the abundance of potentially bioactive compounds in onion waste, their utilization for the production of a variety of beneficial and economically valuable products is very important for both onion producers and processors.

Pectin, a natural complex heteropolysaccharide found in the cell walls of fruits and vegetables, is categorized into two groups: high methoxyl (HMP) and low methoxyl (LMP) pectins, depending on their esterification degree. Commercial pectin is traditionally obtained from citrus peels, apple pulps, sugar beet pulps, and by‐products of juice. Pectin is widely used in a variety of fields, including gelling agents in jams and jellies, stabilizers, and thickeners in fruit juices, the delivery of bioactive compounds, and biomedical applications such as wound healing and tissue engineering (Dranca & Oroian, 2018; Jiang et al., 2020; Naqash et al., 2017; Rehman et al., 2019). In addition to that, the U.S. Food and Drug Administration approves pectin as a safe ingredient for food production. Due to the high demand for pectin in the market, it was estimated that the annual growth rate of pectin reached a compound annual growth rate of 6.5% in 2019 and that its global market value would reach 1.5 billion USD by 2025 (“Pectin Market Report,” 2019).

Various methods for the extraction of pectin have been investigated in order to increase pectin yield and reduce extraction duration and cost. The traditional and most widely used pectin extraction is achieved using the HAE method, carried out at high temperatures under acidic media such as nitric, citric (CA), hydrochloric (HCl), acetic (AA), and sulfuric acids (Koubala et al., 2008). In addition to the HAE technique, a variety of extraction methods, including microwave extraction, enzymatic extraction, ultrasound‐assisted extraction, and ultrasound‐assisted subcritical water extraction, have been developed to obtain better extraction yields and higher quality pectin (Chen et al., 2015; González‐Centeno et al., 2014; Thirugnanasambandham et al., 2014). Organic acids used in the HAE method mainly result in lower dissociation constants, hydrolysis capacities, and pectin yields compared with the utilization of inorganic acids (Picot‐Allain et al., 2022). Previously, pectin extraction was successfully accomplished with a variety of agricultural by‐products (fruit and vegetable waste) using the HAE method under different organic and inorganic acidic media (Şen et al., 2021; Sen & Uguzdogan, 2022). In our previous study, utilization of dual acid mixtures containing organic and inorganic acids for the extraction of pectin from garlic waste provided better yields and good control on the physical properties of extracted pectin from garlic waste (Şen et al., 2022).

In this study, we aim to investigate the extraction of pectin from onion waste using two different extraction methods, PUAE and HAE extractions, under pure water, organic–inorganic acids, or acid mixtures, and compare the yields and physical properties of extracted pectins from these two techniques. The effects of the extraction pH and the kinds of the acid mixture on the yield and quality of the extracted pectin from onion waste were investigated to determine the optimum extraction conditions for both methods. The equivalent weight (EW), degree of esterification (DE), methoxyl (MeO), and galacturonic acid (Gal‐A) contents of the extracted pectin samples were also determined for all extracted samples. Sequential PUAE and HAE methods for the extraction of pectin from onion waste were also investigated in order to increase the yield and improve the physicochemical properties of pectin. Utilization of sequential methods provided highly enhanced pectin yields and better physicochemical properties.

## 2 MATERIALS AND METHODS

### 2.1 Hot‐acid extraction of pectin from onion waste

Conventional extraction of pectin is achieved under the HAE method. In this study, pure water, two organic, two inorganic, and six different mixtures of these acids were used during the extraction processes with a 1/30 (g/mL) solid–liquid ratio (SLR). The organic acids used were CA and AA acids, whereas the inorganic acids were sulfuric (H_2_SO_4_) and HCl acids. Concentrations of acids were 0.1 N. Six gram of dried onion waste powder was poured into 180 mL of extractants for the extraction experiments. The extraction temperature and duration for all experimental conditions were kept at 90°C for 90 min. After completion of the extraction procedure, the solution was filtered to remove residue. Then, pectin was precipitated by the addition of 96% ethanol into the filtrate. The obtained mixture was stored in a refrigerator for 12 h to complete precipitation. The pectin product was then centrifuged and washed four times with ethanol in order to remove contaminants. A NÜVE NF 400 centrifuge (Türkiye) was used during the extraction procedure. Collected pectin in gel form was then dried at room temperature (RT) and stored at 4°C in the refrigerator for further characterization procedures (Şen et al., 2022).

### 2.2 Pulsed ultrasound‐assisted extraction of pectin from onion waste

Extraction of pectin from onion waste was also accomplished using the pulsed ultrasound‐assisted extraction (PUAE) method. PUAE consumes less energy and, therefore, provides power for each bubble and increases their energy storage capacity (Spinei & Oroian, 2022). The optimum extraction condition was detected under 1/30 SLR. Then, the solution was ultrasonicated with pulses (the pulse cycle was 10 s on and 5 s off) at RT for 15 min at 50% amplitude (20 kHz, ultrasonic power of 30–150 W) using an ultrasonic homogenizer Bandelin HD‐4100 (BANDELIN electronic GmbH & Co. KG, Heinrichstrasse 3–4 Germany, Probe:TS113). The extraction mixture was also stirred with a magnetic stirrer to ensure effective extraction during the ultrasonication. Pure water, dilute acid solutions (AA, CA, HCl, and H_2_SO_4_), and different mixtures of these acid solutions were used for the extraction of pectin from onion waste.

### 2.3 Sequential application of PUAE and HAE methods for the extraction of pectin from onion waste

In order to increase the yield and improve the physicochemical properties of pectin, sequential PUAE and HAE extraction methods were applied in the extraction of pectin from onion waste. In this method, extraction was first carried out under PUAE conditions for 15 min at RT with 10 s on and 5 s off pulse cycles. After that, the obtained mixture was subsequently subjected to the HAE method at 90°C for 75 min. After these extraction processes, the purification steps for the extracted pectins were the same as for HAE, as mentioned above.

### 2.4 Extracted pectin yields and physicochemical properties

The extracted pectin yield (EPY) was calculated according to the below equation, where P is the weight of the dried pectin and DO is the weight of the dried onion waste.

$$\mathrm{EPY}\;\left(\%\right)=\frac{P\;\left(g\right)}{\mathrm{DO}\;\left(g\right)}\times 100$$

The Gal‐A contents of the extracted pectins were determined by using the spectrophotometric method developed by Blumenkrantz and Asboe‐Hansen (1973; Sen & Uguzdogan, 2022; Şen et al., 2022). The potentiometric titration method (Bochek et al., 2001) was used to determine the DE and MeO contents of the extracted pectins, and the titrimetric method (Ranganna, 1986) was used to determine the EWs. Experimental protocols to determine the physicochemical properties of the extracted pectins were performed in triplicate. FT‐IR analyses of extracted pectin samples were recorded on a Thermo Fisher Scientific‐Nicolet/IS50 (USA) infrared spectrophotometer device with a diamond ATR probe between 400 and 4000 cm^−1^. The statistical analysis results were given as the mean standard deviation. The data were analyzed using a single‐factor analysis of variance (ANOVA). The data were tested at a 95% confidence interval for statistical significance.

## 3 RESULTS AND DISCUSSION

**FIGURE 1:**
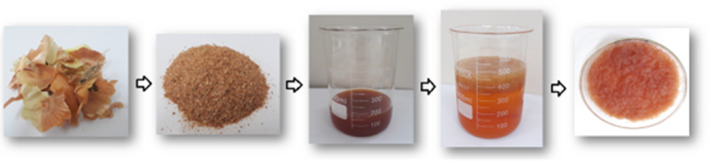
Pectin extraction steps from dried onion waste.

Pectin extraction steps from dried onion waste are shown in Figure 1. Before the extraction procedure, the moisture and ash percentages of onion waste were calculated according to the reported procedure (Şen et al., 2022). The moisture and ash percentages of dried onion waste were respectively found to be 10.97 ± 0.21 and 7.86 ± 0.10%.

Traditional extraction of pectin from different plant sources is mainly achieved by the HAE procedure. PUAE as an advanced extraction technique was also known to be an efficient method for the observation of better yields and physicochemical properties (Tran et al., 2021). PUAE provides moderate heat application to the extracted sample with repetitive on and off modes of the ultrasound process and thereby prevents degradation of bioactive components in the sample (Wani & Uppaluri, 2022a). PUAE can also promote increased mass transfer from the cell walls of plants and reduce the size of particles (Wani & Uppaluri, 2022a). In this study, the effects of both HAE and PUAE methods for the extraction of pectin from onion waste were investigated. Dried onion waste was first ground before the extraction procedure. Sieve analysis of the grounded onion waste was accomplished, and the mass mean diameter of onion powders was calculated to be 356.05 μm (Figure S1). SLR was fixed at 1/30 g/mL according to the reported optimization study (Wani & Uppaluri, 2022b). Extraction media with 1/30 SLR consisted of either pure water and organic–inorganic acids used alone or their mixtures. The organic acids used were CA and AA, whereas the inorganic acids were H_2_SO_4_ and HCl acids. The extraction temperature and duration for all experimental conditions carried out with the HAE method were kept at 90°C for 90 min, as stated in the previous optimization study (Sen & Uguzdogan, 2022). The pH of the acidic extraction medium is significant for the observation of high‐yielded pectin extraction. Lower pH conditions during the extraction process allow isolating more pectin due to its enhanced solubility in strong acidic media (Sarı & Birlik, 2020). Initial and final pH values of the extraction media for both methods are summarized in Table S1. Lower pH conditions allow releasing more pectin products from the plant matrix due to the hydrolysis of cellulose (Pasandide et al., 2018). During the extraction experiments carried out in inorganic acids, the best result in terms of the highest yield was obtained with the condition of H_2_SO_4_ used alone for both PUAE and HAE methods (Table 1). Extractions performed under organic acid conditions (CA and AA) yielded less pectin due to the poor acidities of these acids. Utilization of dual acid mixtures greatly improved extraction yields, especially compared with conditions where organic acids are used alone under HAE method. Increasing inorganic acid concentrations in the extraction media under dual acid conditions resulted in enhanced yields of pectin products (Table 1). For all experimental processes, the initial and final viscosity values of the extraction media were also measured at 21°C (Table S2). The increase in viscosity values at the end of the extraction procedure proves the isolation of pectin from onion waste. A linear relationship was also established between the viscosities of the extraction media and the isolated pectin yields.

**TABLE 1:** Extraction of pectin from onion waste under traditional HAE and PUAE methods.

Entry	Extractant	Volume ratio (V1/V2)	Yields under HAE (%)	Yields under PUAE (%)
1	H_2_O	–	3.57	3.32
2	CA	–	4.53	3.48
3	AA	–	3.57	3.67
4	H_2_SO_4_	–	16.22	9.83
5	HCl	–	10.56	4.83
6	CA‐HCl	3/1	5.52	3.13
7	CA‐HCl	1/1	8.48	3.55
8	CA‐HCl	1/3	9.62	4.80
9	CA‐H_2_SO_4_	3/1	8.22	6.62
10	CA‐H_2_SO_4_	1/1	12.55	8.02
11	CA‐H_2_SO_4_	1/3	15.78	9.22
12	AA‐CA	3/1	3.83	3.63
13	AA‐CA	1/1	4.03	3.44
14	AA‐CA	1/3	4.48	3.30
15	AA‐H_2_SO_4_	3/1	6.93	6.00
16	AA‐H_2_SO_4_	1/1	11.11	7.82
17	AA‐H_2_SO_4_	1/3	15.97	8.75
18	AA‐HCl	3/1	4.73	3.07
19	AA‐HCl	1/1	9.30	3.40
20	AA‐HCl	1/3	10.55	4.53
21	HCl‐H_2_SO_4_	3/1	13.03	7.52
22	HCl‐H_2_SO_4_	1/1	14.13	9.67
23	HCl‐H_2_SO_4_	1/3	15.07	9.82

*Note*: V1: The volume of the first acid in the mixture; V2: The volume of the second acid in the mixture.

According to Table 1, the yields of the extracted pectin samples under HAE conditions were detected to be higher compared to the conditions of the PUAE method. While pectin yields from onion waste under HAE conditions ranged between 3.57% and 16.22%, yields were found to be between 3.32% and 9.83% under the PUAE procedure (Table 1). Previous reports showed that pectin yield from the extraction of onion waste was 9.2% and 7.1%, respectively, for white and red onion under ammonium oxalate conditions at 95°C (Alexander & Sulebele, 1973). Compared to these results, our findings for HAE experiments carried out at 90°C showed better pectin yields from onion waste. The initial solution temperature of the extraction media for the PUAE condition was in the range of 21.4–25.5°C (RT). Extraction temperature is significant for the achievement of the release of pectin from the insoluble parts through hydrolysis (Grassino et al., 2016; Nguyen & Pirak, 2019). Therefore, HAE experiments performed at higher temperatures resulted in higher yielding pectin products compared with the conditions of the PUAE method. After 15 min of extraction under PUAE, the final solution temperature was detected to be around 42.2–46.3°C. Initial and final solution temperatures during the PUAE method and consumed energy values were summarized in Table S3. According to Table S3, the consumed energy values for the extractions carried out under PUAE conditions were in the range of 36.4–39.9 kJ. This value was determined to be 2200 kJ for the HAE method carried out at 90°C for a 90 min extraction duration. Although the extraction yields of the HAE method were higher than those of PUAE, it requires approximately 60 times more energy than the conditions of PUAE in 15 min.

The DE and MeO contents of the obtained pectin samples from both HAE and PUAE methods are given in Table 2. While the ratio of the esterified Gal‐A to the total Gal‐A groups of the pectin samples is defined as the DE, the total number of moles of methanol per 100 mol of Gal‐A is known to be the MeO content (Şen et al., 2022). DE value determines the commercial utilization of pectin, and increasing DE value shows the gelation properties of pectin samples (Şen et al., 2022). According to the obtained results, while the DE and MeO contents of the extracted pectin samples under the HAE method were higher in the conditions carried out with organic acids, these values were found to be higher in the conditions of the PUAE method carried out with inorganic acids. The DE and MeO contents of pectins extracted under dual acid mixtures showed that these values were mostly lower for organic acid mixtures under HAE conditions compared to the PUAE. While the DE value of the commercial pectin was found to be 73.20 ± 0.38% (Table 2), these values of extracted pectin samples under HAE conditions were in the range of 42.86 ± 0.42–62.96 ± 0.55. The highest DE value of 62.96 ± 0.55% was observed in the HAE method carried out under the AA condition (entry 26 in Table 2). The DE contents of the pectin samples extracted with PUAE methods varied between 29.58 ± 0.52 and 63.54 ± 0.66%. Extractions carried out under H_2_SO_4_ condition for the PUAE method resulted in the highest DE content with 63.54 ± 0.66% (entry 27 in Table 2). Interestingly, utilization of organic–inorganic acid mixtures as extraction media resulted in moderate DE values of the pectin samples. As an example, while the DE of the extracted pectin in CA (entry 25 in Table 2) was 60.32 ± 0.39%, extraction conditions carried out with the mixture of CA‐HCl (entry 29–31 in Table 2) resulted in decreasing DE values with increasing HCl concentration. The possible degradation of pectic acid chains and demethylation with increasing acidity could be the main reason for this investigation (Şen et al., 2022). This observation was also detected for all tested dual acid conditions containing organic–inorganic acids. Another interesting result was observed in Table 2; while the DE and MeO values of pectin samples extracted from organic acid conditions with the HAE method were higher, these values were found to be higher for pectins extracted from inorganic acids with the PUAE method. For acid mixtures, DE and MeO values of pectin samples from the HAE method were generally found to be higher with increasing organic acid content than those of the PUAE method (Table 2).

**TABLE 2:** Degree of esterification (DE) and methoxyl content (MeO) of the extracted pectin from onion waste under HAE and PUAE methods.

Entry	Extractant	Volume ratio (V1/V2)	DE under HAE (%)	DE under PUAE (%)	MeO under HAE (%)	MeO under PUAE (%)
*Commercial pectin (CP)*			73.20 ± 0.38^a^	73.20 ± 0.38^a^	12.18 ± 0.25^a^	12.18 ± 0.25^a^
24	H_2_O	–	42.86 ± 0.42^iA^	29.58 ± 0.52^kB^	7.30 ± 0.23^cA^	5.09 ± 0.39^cB^
25	CA	–	60.32 ± 0.39^cA^	50.05 ± 0.77^gB^	10.14 ± 0.41^bA^	8.48 ± 0.58^bB^
26	AA	–	62.96 ± 0.55^bA^	37.37 ± 0.42^jB^	10.56 ± 0.32^bA^	6.39 ± 0.45^bB^
27	H_2_SO_4_	–	47.06 ± 0.73^hB^	63.54 ± 0.66^bA^	7.99 ± 0.56^bB^	10.65 ± 0.50^bA^
28	HCl	–	48.14 ± 0.33^hB^	56.62 ± 0.35^dA^	8.17 ± 0.39^bB^	9.54 ± 0.36^bA^
29	CA–HCl	3/1	56.16 ± 0.44^eA^	52.61 ± 0.26^fA^	9.47 ± 0.69^bA^	8.89 ± 0.58^bA^
30	CA–HCl	1/1	52.55 ± 0.71^fA^	53.16 ± 0.43^eA^	8.88 ± 0.55^bA^	8.98 ± 0.51^bA^
31	CA–HCl	1/3	50.05 ± 0.32^gB^	56.14 ± 0.36^dA^	8.48 ± 0.51^bB^	9.47 ± 0.41^bA^
32	CA–H_2_SO_4_	3/1	56.28 ± 0.29^eA^	54.05 ± 0.42^eB^	9.49 ± 0.52^bA^	9.13 ± 0.38^bA^
33	CA–H_2_SO_4_	1/1	51.46 ± 0.57^gB^	57.35 ± 0.63^cA^	8.71 ± 0.38^bB^	9.66 ± 0.37^bA^
34	CA–H_2_SO_4_	1/3	49.83 ± 0.35^gB^	59.36 ± 0.65^cA^	8.44 ± 0.49^bA^	9.98 ± 0.40^bA^
35	AA–CA	3/1	60.00 ± 0.78^cA^	39.93 ± 0.89^iB^	10.09 ± 0.62^bA^	6.82 ± 0.52^bB^
36	AA–CA	1/1	58.33 ± 0.55^dA^	44.09 ± 0.64^hB^	9.82 ± 0.41^bA^	7.50 ± 0.58^bB^
37	AA–CA	1/3	57.89 ± 0.59^dA^	48.35 ± 0.72^gB^	9.75 ± 0.45^bA^	8.20 ± 0.67^bB^
38	AA–H_2_SO_4_	3/1	61.71 ± 0.54^bA^	43.16 ± 0.59^hB^	10.36 ± 0.66^bA^	7.35 ± 0.45^bB^
39	AA–H_2_SO_4_	1/1	52.63 ± 0.68^fA^	53.81 ± 0.73^eA^	8.90 ± 0.47^bA^	9.09 ± 0.48^bA^
40	AA–H_2_SO_4_	1/3	50.06 ± 0.42^gB^	58.35 ± 0.52^cA^	8.48 ± 0.56^bB^	9.82 ± 0.68^bA^
41	AA–HCl	3/1	62.16 ± 0.36^bA^	41.85 ± 0.88^iB^	10.43 ± 0.41^bA^	7.13 ± 0.35^bB^
42	AA–HCl	1/1	53.06 ± 0.47^fA^	50.63 ± 0.73^gB^	8.97 ± 0.40^bA^	8.57 ± 0.39^bA^
43	AA–HCl	1/3	50.94 ± 0.23^gB^	55.45 ± 0.68^dA^	8.62 ± 0.43^bA^	9.35 ± 0.46^bA^
44	HCl–H_2_SO_4_	3/1	47.90 ± 0.58^hB^	58.32 ± 0.42^cA^	8.13 ± 0.55^bB^	9.82 ± 0.48^bA^
45	HCl–H_2_SO_4_	1/1	47.43 ± 0.42^hB^	59.88 ± 0.63^cA^	8.05 ± 0.59^bB^	10.07 ± 0.39^bA^
46	HCl–H_2_SO_4_	1/3	46.94 ± 0.67^hB^	61.15 ± 0.69^cA^	7.97 ± 0.65^bB^	10.27 ± 0.41^bA^

*Note*: V1: The volume of the first acid in the mixture; V2: The volume of the second acid in the mixture. Results were given as mean ± standard deviation. Averages marked with different letters (in the same row A and B and in the same column a, b, c, d, e, f, g, h, i, j, and k) are statistically different from each other (*p* < .05).

EWs and Gal‐A contents of the extracted pectin from onion waste under HAE and PUAE methods are summarized in Table 3. The Gal‐A content of commercial pectin was higher than that of extracted pectin samples from both HAE and PUAE methods. Traditionally, the Gal‐A content of a pectin product is expected to be higher than 65% for commercial applications. The Gal‐A content of the commercial pectin was discovered to be 82.10%. Pectin should be further purified if the Gal‐A content is lower than 65%. According to Table 3, extracted pectin from both HAE and PUAE methods showed lower Gal‐A contents below 65%. Therefore, further purification steps should be applied to achieve more accepted values for the Gal‐A contents of the obtained pectin samples for commercial applications (He et al., 2021). According to Table 3, the Gal‐A contents of the obtained pectin products were found to be higher with increasing inorganic acid contents of dual acid extraction media.

**TABLE 3:** Equivalent weights (EW) and galacturonic acid (Gal‐A) contents of the extracted pectins from onion waste under HAE and PUAE methods.

Entry	Extractant	Volume ratio (V1/V2)	EW under HAE (g/mol)	EW under PUAE (g/mol)	Gal‐A under HAE (%)	Gal‐A under PUAE (%)
*Commercial pectin (CP)*			1000 ± 6.33^i^	1000 ± 6.33^d^	82.10 ± 0.52^a^	82.10 ± 0.52^a^
47	H_2_O	‐	1667 ± 6.23^eA^	883 ± 5.83^gB^	38.97 ± 0.63^gA^	31.81 ± 0.55^jB^
48	CA	‐	1181 ± 5.41^gA^	556 ± 5.48^oB^	37.82 ± 0.59^gA^	27.03 ± 0.52^jB^
49	AA	‐	3333 ± 7.11^aA^	736 ± 6.23^jB^	34.21 ± 0.45^hB^	37.91 ± 0.55^gA^
50	H_2_SO_4_	‐	690 ± 6.49^oB^	1068 ± 5.88^aA^	43.85 ± 0.48^fA^	30.69 ± 0.59^jB^
51	HCl	‐	645 ± 6.55^pB^	734 ± 5.36^jA^	55.12 ± 0.68^bA^	50.79 ± 0.53^bB^
52	CA‐HCl	3/1	1050 ± 5.33^hA^	638 ± 7.22^mB^	42.51 ± 0.71^fA^	33.73 ± 0.47^iB^
53	CA‐HCl	1/1	890 ± 6.12^kA^	676 ± 6.82^lB^	49.38 ± 0.55^dA^	44.65 ± 0.51^eB^
54	CA‐HCl	1/3	715 ± 6.18^nA^	700 ± 5.96^kB^	54.07 ± 0.58^bA^	49.33 ± 0.50^cB^
55	CA‐H_2_SO_4_	3/1	1050 ± 5.38^hA^	853 ± .31^hB^	38.82 ± 0.59^gA^	28.27 ± 0.67^jB^
56	CA‐H_2_SO_4_	1/1	885 ± 6.93^kB^	978 ± 5.99^eA^	40.81 ± 0.62^fA^	29.11 ± 0.62^jB^
57	CA‐H_2_SO_4_	1/3	734 ± 4.48^mB^	1042 ± 5.87^bA^	42.90 ± 0.52^fA^	29.88 ± 0.58^jB^
58	AA‐CA	3/1	2000 ± 5.41^bA^	605 ± 6.18^nB^	35.69 ± 0.51^gA^	30.98 ± 0.53^jB^
59	AA‐CA	1/1	1667 ± 5.68^eA^	667 ± 6.69^lB^	36.21 ± 0.67^gA^	32.63 ± 0.69^jB^
60	AA‐CA	1/3	1250 ± 5.43^fA^	727 ± 5.66^jB^	37.55 ± 0.62^gA^	36.13 ± 0.58^hA^
61	AA‐H_2_SO_4_	3/1	1967 ± 5.97^cA^	839 ± 7.10^hB^	35.56 ± 0.44^gA^	34.96 ± 0.61^iA^
62	AA‐H_2_SO_4_	1/1	1050 ± 4.98^hA^	976 ± 5.23^eB^	36.73 ± 0.48^gB^	32.88 ± 0.59^jB^
63	AA‐H_2_SO_4_	1/3	790 ± 6.28^lB^	1040 ± 5.25^bA^	41.68 ± 0.43^fB^	31.45 ± 0.49^jB^
64	AA‐HCl	3/1	1767 ± 6.11^dA^	732 ± 6.42^jB^	40.95 ± 0.59^fA^	40.30 ± 0.55^fA^
65	AA‐HCl	1/1	969 ± 5.81^jA^	740 ± 6.51^jB^	51.38 ± 0.51^cA^	46.07 ± 0.69^dB^
66	AA‐HCl	1/3	725 ± 6.25^mA^	735 ± 6.33^jA^	54.79 ± 0.52^bA^	48.84 ± 0.58^cB^
67	HCl‐H_2_SO_4_	3/1	655 ± 5.89^pB^	825 ± 6.33^iA^	49.38 ± 0.58^dA^	48.59 ± 0.54^cA^
68	HCl‐H_2_SO_4_	1/1	675 ± 6.26^oB^	942 ± 6.38^fA^	47.71 ± 0.63^eA^	40.28 ± 0.53^fB^
69	HCl‐H_2_SO_4_	1/3	685 ± 5.93^oB^	1020 ± 6.23^cA^	44.08 ± 0.49^fA^	34.01 ± 0.59^iB^

*Note*: V1: The volume of the first acid in the mixture; V2: The volume of the second acid in the mixture. Results were given as mean ± standard deviation. Averages marked with different letters (in the same row A and B and in the same column a, b, c, d, e, f, g, h, i, j, and k) are statistically different from each other (*p* < .05).

The EW values of the extracted pectin samples from onion waste were also summarized in Table 3. Unesterified Gal‐A units and the gel‐forming ability of the pectin samples could be determined by looking at the EW values. Utilization of highly acidic solutions, such as H_2_SO_4_ and HCl, resulted in lower EW values for both methods due to the degradation of pectin chains under lower pH conditions (Table 3). These observations were detected for both the PUAE and HAE methods.

After these observations, extraction of pectin from onion waste was also accomplished under sequential PUAE and HAE methods where the conditions of organic or inorganic acids used alone (Table 4). Extractions under different acidic media were first performed under ultrasonication at RT (PUAE) in 15 min and then at 90°C in 75 min under HAE. Table S4 also shows consumed energy values for the extractions carried out under sequential PUAE and HAE methods (Supporting Information). Consumed energy values were found to be approximately 1872 kJ, which is 15% lower than those achieved under HAE conditions. Utilization of sequential methods provided enhanced pectin yields up to 20.32% (entry 73 in Table 4) compared with the conditions where these methods were used alone. Especially, in combination of PUAE with HAE technique, physical properties of the obtained pectin samples were slightly improved compared with the conditions the PUAE method used alone. For example, while the Gal‐A content of the pectin sample extracted under the PUAE method (entry 51 in Table 3) was 50.79 ± 0.53%, it was found to be 61.70 ± 0.51% in sequential PUAE and HAE methods (entry 74 in Table 4). The DE and MeO contents of all obtained samples were found to be higher compared to the conditions carried out with PUAE alone. Some of these values were also observed to be higher than the conditions of HAE. For example, extractions carried out in HCl solution with sequential HAE and PUAE methods (entry 74 in Table 4) showed higher DE and MeO contents than the conditions of HAE used alone (entry 28 in Table 2).

**TABLE 4:** Extraction of pectin from onion waste under sequential PUAE (15 min) and HAE (75 min) methods.^1^

Entry	Extractant	Yields (%)	DE (%)	MeO (%)	EW (g/mol)	Gal‐A (%)
70	H_2_O	5.13	42.86 ± 0.44^d^	7.30 ± 0.39^b^	1667 ± 5.88^b^	36.97 ± 0.45^d^
71	CA	7.23	61.90 ± 0.51^a^	10.39 ± 0.53^a^	1250 ± 5.69^c^	36.39 ± 0.42^d^
72	AA	5.48	62.50 ± 0.42^a^	10.49 ± 0.48^a^	2500 ± 6.82^a^	40.06 ± 0.43^c^
73	H_2_SO_4_	20.32	46.15 ± 0.47^c^	7.84 ± 0.61^b^	769 ± 6.12^d^	45.88 ± 0.52^b^
74	HCl	14.70	50.00 ± 0.35^b^	8.47 ± 0.58^b^	625 ± 6.72^e^	61.70 ± 0.51^a^

^1^ Extractions were carried out with a 1/30 SLR. PUAE experiments were performed at 50% amplitude, and the pulse cycle was 10 s on and 5 s off. Results were given as mean ± standard deviation. Means marked with different letters in the same column (a, b, c, d, and e) are statistically different from each other (*p* < .05).

In order to compare the effect of applying higher extraction temperatures for the PUAE method, two different experiments with H_2_SO_4_ as an extractant were carried out at 80 and 90°C, with other variables kept the same (Table S5). According to the obtained results, extraction yields for PUAE conditions were highly improved by applying higher temperatures. The obtained pectin yield for the extraction condition carried out under PUAE at 90°C was 18.15% (entry 86 in Table S5), which was higher than the yield (16.22%) of the same experiment carried out under the HAE method (entry 4 in Table 1). The same experimental condition carried out under PUAE conditions at RT (21.5°C) resulted in a 9.83% yield of pectin (entry 4 in Table 1). As mentioned above, this result is due to the increasing release of pectin from the insoluble parts of onion waste at higher temperatures (Grassino et al., 2016; Nguyen & Pirak, 2019). Consumed energy value also enhanced with increasing extraction temperature, and this value was calculated to be 288 kJ for the PUAE method carried out at 90°C in a 15 min extraction duration (entry 86 in Table S5), which was about eight times higher compared with the conditions carried out at RT (entry 60 in Table S3). Even though the yield of pectin product was high under the PUAE condition at 90°C, the physicochemical parameters of the obtained pectin product, including DE, MeO, and EW values, were found to be lower compared with those of the conditions of entry 2 in Table 1.

**FIGURE 2:**
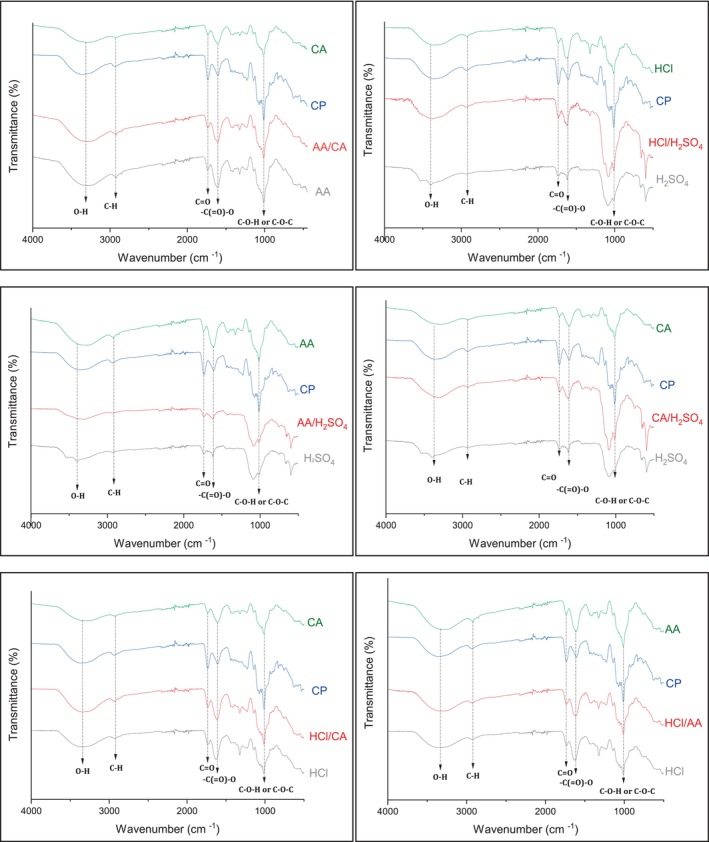
FT‐IR spectra of commercial pectin (CP) and pectin samples extracted from onion waste in different acids or acid mixtures (1/1 V/V) under the HAE method.

FT‐IR spectra results of the extracted pectin samples and commercial pectin from citrus are illustrated in Figure 2. Each FT‐IR spectrum shows extracted pectins from onion waste under either an acid or acid mixture (1/1 V/V). According to the obtained FT‐IR spectra results, extracted pectin samples showed similar vibration bands compared to those of commercial pectin from citrus. The existence of characteristic carbonyl (C=O) and carboxylate (COO) functional group vibration bands was observed around 1750 and 1600 cm^−1^, respectively. Peaks appearing at 3400 and 2900 cm^−1^ were attributed to the respective hydroxyl and C–H stretching vibrations of the pectin samples. The band that appeared around 1000 cm^−1^ corresponded to the C–O stretching vibration band (Kallel et al., 2015). The obtained FT‐IR data confirmed the pectin structures of the extracted samples from onion waste.

## 4 CONCLUSIONS

In this study, a comprehensive comparison between HAE and PUAE methods for the extraction of pectin from onion waste was introduced under different organic–inorganic acids and their mixtures. The extraction temperature for all experimental conditions carried out under the HAE method was kept at 90°C, whereas PUAE experiments were accomplished at RT. In general, the HAE method gave better pectin yields compared with the conditions of PUAE used alone due to the significance of the increasing extraction temperature for the release of pectin from the plant matrix. The PUAE method provides less time‐ and energy‐consuming extraction of pectin from onion waste within 15 min at RT and thus seems to be more economic compared with the HAE method. The physicochemical properties (EW, MeO, and DE contents) of the obtained pectins indicated that isolated pectins were mostly HMP. Sequential HAE and PUAE methods for the extraction of pectin from onion waste were also found to be very useful in terms of obtaining higher yields and better physicochemical properties. Extracted pectin products by both HAE and PUAE methods were also analyzed by FT‐IR spectroscopy. FT‐IR analyses showed that extracted products showed similar vibration bands compared with those of commercial citrus pectin. Further studies should be focused on altering the purification steps of isolated pectin for better purity and evaluating extracted pectin products for different industrial purposes, including biofuel and bioenergy.

## AUTHOR CONTRIBUTIONS

**Emine Şen:** Formal analysis (equal); investigation (equal); validation (equal); visualization (equal). **Ersen Göktürk:** Writing – original draft (lead); writing – review and editing (lead). **Vugar Hajiyev:** Investigation (equal); validation (equal). **Erdal Uğuzdoğan:** Conceptualization (lead); methodology (lead); resources (lead); validation (equal); writing – original draft (equal); writing – review and editing (equal).

## CONFLICT OF INTEREST STATEMENT

The authors have no competing interests to declare that are relevant to the content of this article.

## COMPLIANCE WITH ETHICS REQUIREMENTS

This article does not contain any studies with human participants or animals performed by any of the authors.

## Supporting information


Data S1

## Data Availability

The data that supports the findings of this study is available in the Supporting Information of this article.
