# Supplementary material for: Comparisons of pulsed ultrasound‐assisted and hot‐acid extraction methods for pectin extraction under dual acid mixtures from onion (Allium cepa L.) waste
Source: Food Sci Nutr. 2023 Sep 7;11(11):7320–9. doi: 10.1002/fsn3.3657 (PMC10630788; doi:10.1002/fsn3.3657)
Supplement: Supplementary file 1 — Data S1 [file FSN3-11-7320-s001.docx]

**Comparisons of pulsed ultrasound-assisted and hot-acid extraction methods for pectin extraction under dual acid mixtures from onion (*Allium cepa L.*) waste**

Emine Şen^a^, Ersen Göktürk^b^, Vugar Hajiyev^a^, Erdal Uğuzdoğan^a^*

^a^Pamukkale University, Department of Chemical Engineering, TR-20070 Denizli, Türkiye

^b^Hatay Mustafa Kemal University, Department of Chemistry, TR-31060 Hatay, Türkiye

*Corresponding author: Erdal UĞUZDOĞAN

E-mail addresses:

Erdal UĞUZDOĞAN

[erdalu@pau.edu.tr](mailto:erdalu@pau.edu.tr)

<https://orcid.org/0000-0003-0607-1304>

Emine ŞEN

[esen162@posta.pau.edu.tr](mailto:esen162@posta.pau.edu.tr)

<https://orcid.org/0000-0001-6244-9880>

Ersen Göktürk

[ersengokturk@gmail.com](mailto:ersengokturk@gmail.com)

<https://orcid.org/0000-0001-6742-2847>

Vugar Hajiyev

[vhajiyev17@posta.pau.edu.tr](mailto:vhajiyev17@posta.pau.edu.tr)

[https://orcid.org/0000-0002-1214-8067](https://orcid.org/0000-0001-6244-9880)

**Materials and Methods**

**Materials**

Citric Acid, hydrochloric acid and ethyl alcohol were purchased from Merck KgaA. Pectin from citrus peel, sodium hydroxide, 3-phenylphenol, D-(+)-galacturonic acid monohydrate and sodium tetraborate were purchased from Sigma Aldrich. Acetic acid and sulfuric acid were purchased from Riedel-de Haen and Isolab, respectively. All other chemicals and reagents were used as analytical grade unless otherwise specified. Macherey-Nagel MN 640w # 41 black ribbon filter paper was used for the experiments. Termal-H11900 shaking water bath (Türkiye) was used during the experimental studies. Onion waste (peels and straw) was obtained from the dining hall of the university.

Onion waste was dried at 50 ºC in an oven (JSON-100 Natural Convection Oven 100L, JS Research Inc., South Korea). Then, dried onion waste was powdered by Retsch S/S Cross Beater Hammer Mill Sk1 milling device. Particle size of the raw materials used in the extraction is one of the important parameters especially for enhancing the yield of extracted pectins. For this reason, the mass average particle size of grounded onion waste was determined. The average particle size of the onion waste powder was determined using sieve analysis using Fritsch–Analysette 3 Spartan shaking sieve system. Eight different sieves ranging from 63 to 850 µm (63, 90, 125, 180, 250, 355, 500 and 850 µm) diameters were used to determine the mean overall diameter (mass mean diameter) of the ground onion waste. The mass mean diameter of the ground onion waste were calculated using the below equation (eq. S1) suggested by Fellows (1). According to the sieve analysis results of the ground onion waste, the mass mean diameter was 356.05 µm (Figure S1).

| $d_{m}=\frac{\sum d}{\sum m}$ | (S1) |
| --- | --- |

The pH value of the extraction medium is very important for the extraction of pectin. Therefore, the pH values of the extractants and extracts were determined, and given in Table S1. HANNA HI 83141 digital pH meter was used to measure solution pH of the extractants and extracts. Viscosities of the extracts were determined before and after extraction media in order to determine whether pectin was released from the onion waste to the solution. Viscosity measurements for the extractant and extract were accomplished using AND Vibro Viscometer SV-10.

**Figure S1.** Sieve analysis results of the ground onion waste.

**Table S1.** pH values of extractants and extracts under different organic-inorganic acids/acid mixtures and in different extraction methods.

| **Entry** | **Extractant** | **Volume ratio (V1/V2)** | **Initial pH** | **Extract pH (HAE)** | **Extract pH (PUAE)** |
| --- | --- | --- | --- | --- | --- |
| 1 | H_2_O | - | 5.44 | 4.40 | 4.44 |
| 2 | CA | - | 2.25 | 3.13 | 3.06 |
| 3 | AA | - | 2.98 | 3.93 | 3.80 |
| 4 | H_2_SO_4_ | - | 1.23 | 1.58 | 1.46 |
| 5 | HCl | - | 1.03 | 1.34 | 1.28 |
| 6 | CA-HCl | 3/1 | 1.64 | 2.74 | 2.55 |
| 7 | CA-HCl | 1/1 | 1.35 | 2.09 | 1.92 |
| 8 | CA-HCl | 1/3 | 1.20 | 1.48 | 1.46 |
| 9 | CA-H_2_SO_4_ | 3/1 | 1.64 | 2.63 | 2.60 |
| 10 | CA-H_2_SO_4_ | 1/1 | 1.43 | 2.14 | 2.02 |
| 11 | CA-H_2_SO_4_ | 1/3 | 1.19 | 1.67 | 1.65 |
| 12 | AA-CA | 3/1 | 2.52 | 3.64 | 3.58 |
| 13 | AA-CA | 1/1 | 2.42 | 3.50 | 3.34 |
| 14 | AA-CA | 1/3 | 2.27 | 3.27 | 3.20 |
| 15 | AA-H_2_SO_4_ | 3/1 | 1.74 | 2.90 | 2.82 |
| 16 | AA-H_2_SO_4_ | 1/1 | 1.46 | 2.20 | 2.03 |
| 17 | AA-H_2_SO_4_ | 1/3 | 1.34 | 1.71 | 1.65 |
| 18 | AA-HCl | 3/1 | 1.60 | 2.92 | 2.90 |
| 19 | AA-HCl | 1/1 | 1.34 | 2.14 | 1.97 |
| 20 | AA-HCl | 1/3 | 1.16 | 1.55 | 1.50 |
| 21 | HCl-H_2_SO_4_ | 3/1 | 1.08 | 1.36 | 1.30 |
| 22 | HCl-H_2_SO_4_ | 1/1 | 1.14 | 1.46 | 1.34 |
| 23 | HCl-H_2_SO_4_ | 1/3 | 1.13 | 1.48 | 1.38 |
| ***15 min PUAE and 75 min HAE*** | | | ***Extractant pH*** | ***Extract pH (PUAE+HAE)*** | |
| 24 | H_2_O | - | 5.44 | 4.30 | |
| 25 | CA | - | 2.25 | 3.04 | |
| 26 | AA | - | 2.98 | 3.76 | |
| 27 | H_2_SO_4_ | - | 1.23 | 1.46 | |
| 28 | HCl | - | 1.03 | 1.26 | |

* HAE: Hot-acid extraction, PUAE: Pulsed ultrasonic assisted extraction, V1: The volume of the first acid, V2: The volume of the second acid.

**Table S2.** Viscosity values of extractants and extracts under different organic-inorganic acids/acid mixtures and in different extraction methods.

| **Entry** | **Extractant** | **Volume ratio (V1/V2)** | **Initial viscosity (cP at 21 ºC)** | **Viscosity of extract for HAE (cP at 21 ºC)** | **Viscosity of extract for PUAE (cP at 21 ºC)** |
| --- | --- | --- | --- | --- | --- |
| 29 | H_2_O | - | 0.81 | 1.27 | 0.94 |
| 30 | CA | - | 0.78 | 1.04 | 1.20 |
| 31 | AA | - | 0.83 | 1.00 | 1.05 |
| 32 | H_2_SO_4_ | - | 0.81 | 1.46 | 1.92 |
| 33 | HCl | - | 0.79 | 1.21 | 1.96 |
| 34 | CA-HCl | 3/1 | 0.82 | 1.25 | 1.13 |
| 35 | CA-HCl | 1/1 | 0.79 | 1.29 | 1.15 |
| 36 | CA-HCl | 1/3 | 0.79 | 1.43 | 1.58 |
| 37 | CA-H_2_SO_4_ | 3/1 | 0.78 | 1.29 | 1.17 |
| 38 | CA-H_2_SO_4_ | 1/1 | 0.78 | 1.43 | 1.20 |
| 39 | CA-H_2_SO_4_ | 1/3 | 0.87 | 1.80 | 1.59 |
| 40 | AA-CA | 3/1 | 0.82 | 1.14 | 1.01 |
| 41 | AA-CA | 1/1 | 0.78 | 1.09 | 1.03 |
| 42 | AA-CA | 1/3 | 0.83 | 1.14 | 1.04 |
| 43 | AA-H_2_SO_4_ | 3/1 | 0.84 | 1.31 | 1.17 |
| 44 | AA-H_2_SO_4_ | 1/1 | 0.80 | 1.34 | 1.26 |
| 45 | AA-H_2_SO_4_ | 1/3 | 0.84 | 1.80 | 1.62 |
| 46 | AA-HCl | 3/1 | 0.84 | 1.29 | 1.08 |
| 47 | AA-HCl | 1/1 | 0.78 | 1.67 | 1.31 |
| 48 | AA-HCl | 1/3 | 0.83 | 1.50 | 1.62 |
| 49 | HCl-H_2_SO_4_ | 3/1 | 0.84 | 1.39 | 1.70 |
| 50 | HCl-H_2_SO_4_ | 1/1 | 0.78 | 1.25 | 2.01 |
| 51 | HCl-H_2_SO_4_ | 1/3 | 0.83 | 1.54 | 2.02 |
| ***15 min UAE and 75 min HAE*** | | | ***Initial viscosity (cP at 21* ºC*)*** | ***Viscosity of extract (PUAE+HAE) (cP at 21* ºC*)*** | |
| 52 | H_2_O | - | 0.81 | 1.13 | |
| 53 | CA | - | 0.78 | 1.21 | |
| 54 | AA | - | 0.83 | 1.07 | |
| 55 | H_2_SO_4_ | - | 0.81 | 1.88 | |
| 56 | HCl | - | 0.79 | 1.70 | |

* HAE: Hot acid extraction, PUAE: Pulsed ultrasonic assisted extraction, V1: The volume of the first acid, V2: The volume of the second acid.

**Table S3.** Initial and final solution temperatures during PUAE method and calculated values of consumed energy.

| **Entry** | **Extractant** | **Volume ratio (V1/V2)** | **Yields under PUAE (%)** | **T_i_ (ºC)** | **T_f_ (ºC)** | **Consumed energy (kJ)** |
| --- | --- | --- | --- | --- | --- | --- |
| 57 | H_2_O | - | 3.32 | 24.4 | 46.0 | 38.4 |
| 58 | CA | - | 3.48 | 21.5 | 42.8 | 36.6 |
| 59 | AA | - | 3.67 | 21.5 | 42.7 | 36.5 |
| 60 | H_2_SO_4_ | - | 9.83 | 21.5 | 42.2 | 36.6 |
| 61 | HCl | - | 4.83 | 21.4 | 43.3 | 36.4 |
| 62 | CA-HCl | 3/1 | 3.13 | 25.5 | 46.2 | 39.3 |
| 63 | CA-HCl | 1/1 | 3.55 | 25.0 | 45.8 | 38.2 |
| 64 | CA-HCl | 1/3 | 4.80 | 25.2 | 46.3 | 38.7 |
| 65 | CA-H_2_SO_4_ | 3/1 | 6.62 | 24.3 | 44.8 | 38.3 |
| 66 | CA-H_2_SO_4_ | 1/1 | 8.02 | 24.4 | 45.3 | 36.5 |
| 67 | CA-H_2_SO_4_ | 1/3 | 9.22 | 24.3 | 45.3 | 37.8 |
| 68 | AA-CA | 3/1 | 3.63 | 24.6 | 46.0 | 39.3 |
| 69 | AA-CA | 1/1 | 3.44 | 23.5 | 45.1 | 38.9 |
| 70 | AA-CA | 1/3 | 3.30 | 23.5 | 45.1 | 39.9 |
| 71 | AA-H_2_SO_4_ | 3/1 | 6.00 | 21.9 | 43.0 | 37.7 |
| 72 | AA-H_2_SO_4_ | 1/1 | 7.82 | 21.7 | 42.9 | 37.9 |
| 73 | AA-H_2_SO_4_ | 1/3 | 8.75 | 21.8 | 42.7 | 37.7 |
| 74 | AA-HCl | 3/1 | 3.07 | 23.7 | 45.3 | 39.0 |
| 75 | AA-HCl | 1/1 | 3.40 | 23.7 | 45.2 | 38.5 |
| 76 | AA-HCl | 1/3 | 4.53 | 23.7 | 45.6 | 38.0 |
| 77 | HCl-H_2_SO_4_ | 3/1 | 7.52 | 24.0 | 44.1 | 36.9 |
| 78 | HCl-H_2_SO_4_ | 1/1 | 9.67 | 24.1 | 45.6 | 39.2 |
| 79 | HCl-H_2_SO_4_ | 1/3 | 9.82 | 23.7 | 45.2 | 38.6 |

* PUAE: Pulsed ultrasonic assisted extraction, V1: The volume of the first acid, V2: The volume of the second acid.

**Table S4.** The amounts of the consumed energy for *15 min PUAE* and *75 min HAE*

| **Entry** | **Extractant** | **PUAE (kJ)** | **HAE (kJ)** | **Total (kJ)** |
| --- | --- | --- | --- | --- |
| 80 | H_2_O | 38.4 | 1835 | 1873.4 |
| 81 | CA | 36.6 | 1835 | 1871.6 |
| 82 | AA | 36.5 | 1835 | 1871.5 |
| 83 | H_2_SO_4_ | 36.6 | 1835 | 1871.6 |
| 84 | HCl | 36.4 | 1835 | 1871.4 |

**Table S5.** Extraction of pectin from onion waste under PUAE condition in H_2_SO_4_ extractant at higher temperatures.

| **Entry** | **Extractant** | **T (ºC)** | **UAE (kJ)** | **pH** | **Yield (%)** | **DE (%)** | **MeO (%)** | **EW (g/mol)** |
| --- | --- | --- | --- | --- | --- | --- | --- | --- |
| 85 | H_2_SO_4_ | 80 | 216 | 1.38 | 15.83 | 45.45 | 7.73 | 714 |
| 86 | H_2_SO_4_ | 90 | 288 | 1.35 | 18.15 | 52.00 | 8.80 | 714 |

*Experiments were carried out with 1/30 SLR in 15 min extraction duration (50% amplitude, the pulse cycle was 10 s on and 5 s off)

**References**

1. Fellows PJ (2017) Food Processing Technology: Principles and practice, 4th Edn. Elsevier, pp. 220-221.
